# Older adults' experience with virtual conversational agents for health data collection

**DOI:** 10.3389/fdgth.2023.1125926

**Published:** 2023-03-15

**Authors:** Hattie Wilczewski, Hiral Soni, Julia Ivanova, Triton Ong, Janelle F. Barrera, Brian E. Bunnell, Brandon M. Welch

**Affiliations:** ^1^ Doxy.me Research, Doxy.me Inc., Rochester, NY, United States; ^2^ Department of Psychiatry and Behavioral Neurosciences, University of South Florida, Tampa, FL, United States; ^3^ Department of Public Health Sciences, Medical University of South Carolina, Charleston, SC, United States

**Keywords:** health data collection, older adults, virtual conversational agents, chatbot, user experience, usability

## Abstract

**Introduction:**

Virtual conversational agents (i.e., chatbots) are an intuitive form of data collection. Understanding older adults' experiences with chatbots could help identify their usability needs. This quality improvement study evaluated older adults' experiences with a chatbot for health data collection. A secondary goal was to understand how perceptions differed based on length of chatbot forms.

**Methods:**

After a demographic survey, participants (≥60 years) completed either a short (21 questions), moderate (30 questions), or long (66 questions) chatbot form. Perceived ease-of-use, usefulness, usability, likelihood to recommend, and cognitive load were measured post-test. Qualitative and quantitative analyses were used.

**Results:**

A total of 260 participants reported on usability and satisfaction metrics including perceived ease-of-use (5.8/7), usefulness (4.7/7), usability (5.4/7), and likelihood to recommend (Net Promoter Score = 0). Cognitive load (12.3/100) was low. There was a statistically significant difference in perceived usefulness between groups, with a significantly higher mean perceived usefulness for Group 1 than Group 3. No other group differences were observed. The chatbot was perceived as quick, easy, and pleasant with concerns about technical issues, privacy, and security. Participants provided suggestions to enhance progress tracking, edit responses, improve readability, and have options to ask questions.

**Discussion:**

Older adults found the chatbot to be easy, useful, and usable. The chatbot required low cognitive load demonstrating it could be an enjoyable health data collection tool for older adults. These results will inform the development of a health data collection chatbot technology.

## 1. Introduction

Health data collection (HDC) (e.g., intake forms, medical history, clinical assessments, etc.) is a critical tool for health care providers to obtain an accurate understanding of health (1). Complete patient data is of utmost importance for older adults as they tend to use health services more than younger patients (2). This tendency may require older adults to complete health forms more frequently, emphasizing the need for easy-to-use HDC approaches to optimize user experiences and yield higher quality health information.

With advancements in healthcare technology and the onset of COVID-19, remote HDC is increasing. Compared to traditional paper-based or in-clinic HDC, remote approaches allow patients to complete health forms at a convenient time and place with easier data input, more flexible corrections, fewer errors, and integration with other health information technologies. In recent years, chatbots have demonstrated better usability and user experience compared to other online HDC tools (3–5). Patients have reported chatbots to be intuitive, engaging, and trustworthy, all of which can contribute to higher quality information sharing and less reactive impression management when collecting health data (6, 7).

Since older adults are often homebound and utilize more healthcare services, it is important that they have access to easy-to-use healthcare services and technologies (2, 8). Yet, older adults are rarely included in the design and development of new health technologies (9). They are more likely to find new tasks to be difficult and blame themselves for poor design problems, and they are less confident navigating online interfaces (9, 10). There is also a need to address older adults' apprehension with health data collection, storage, and usage (11). Although older adults tend to have positive attitudes towards the benefits of new health technologies, they are often slow to adopt and engage with such technologies (8, 11, 12). Previous research with older adults has concluded chatbots present an improved user experience for HDC, reducing workload by presenting questions one at a time and mimicking a friendly dialogue (8, 13). These studies acknowledged the need for better understanding of the user experience of HDC *via* chatbots among older adults.

The purpose of this quality improvement (QI) study was to evaluate the workload, usability, and ease-of-use of a chatbot-delivered HDC among older adults. Considering patients are often required to complete long or multiple health forms, we aimed to understand whether the length (measured as numbers of questions) of three chatbot-delivered health forms impacted older adults’ perceptions.

## 2. Materials and methods

### 2.1. Study settings and participants

**Figure 1:**
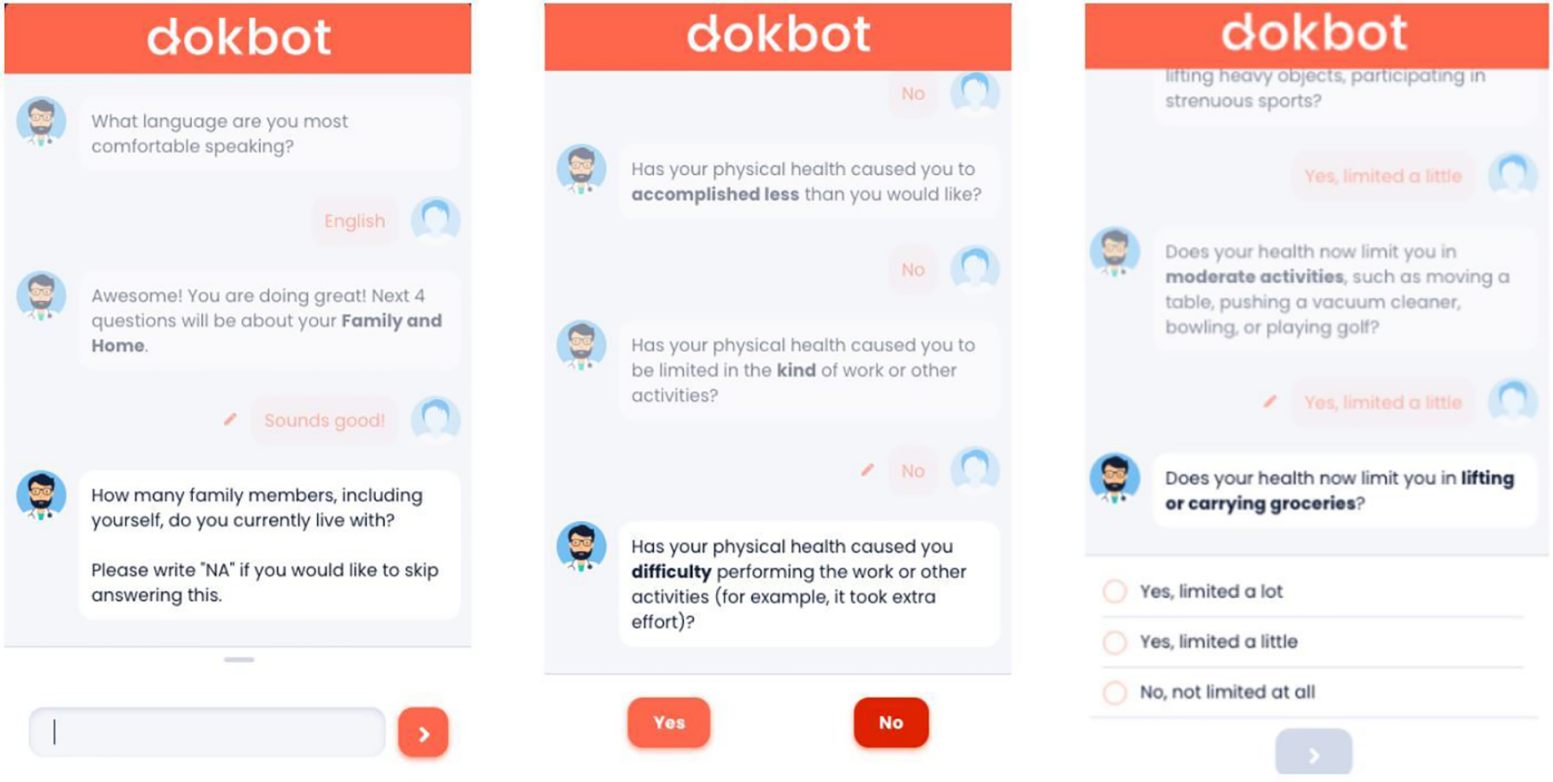
Chatbot interface showing various questions and response types.

In this study, older adults' experiences with data collection were assessed using a chatbot, Dokbot^1^ (14). Dokbot is a free, simple, web-based, and HIPAA-compliant chatbot designed for HDC. It mimics human-to-human interaction by using a mobile, chat-based, interactive approach and can be customized with various names, avatars, languages, and personalities appropriate to end-user characteristics (e.g., age, sex). Dokbot's customizable contrast ratio of font to background ensures higher visibility for users, and changes in size of font are available through users' phone, tablet, or computer since it is web-based. Dokbot can be integrated within different health information technology systems and websites. Figure 1 displays screenshots of the chatbot interface for different question types.

Eligible participants for this study included older adults based in the United States aged ≥60 years old. The age range for older adults was chosen based on previous research on assessing older adults' experience with chatbots and health data collection (8, 15). We aimed to recruit 300 participants, with 100 participants in each of three groups. The three groups differed in length of health forms presented by the chatbot (i.e., short, moderate, and long). Participants were recruited through Prolific^2^, an online crowdsourcing platform with a history of high data quality (16, 17). Prolific participants are assigned a unique 24-character alphanumeric code (Prolific ID) to link their responses and successful payment. Between March 7 and 8, 2022 a total of 353 participants based in the United States were screened for age (≥60 years old). We identified a group of 334 participants who met our age criteria to accrue the final 300 participants. Participants were paid $0.16 to complete the screener. Using Microsoft Excel, participants were randomly divided into three groups: Group 1 (short form; *n* = 113), Group 2 (moderate form; *n* = 111), and Group 3 (long form; *n* = 110). Section 2.3. provides details about the groups. Group 1 recruitment took place March 8–17, 2022; Group 2 and Group 3 recruitment took place March 21–April 4, 2022. Participants were compensated either $2.00 (Group 1), $2.25 (Group 2), or $2.50 (Group 3) for participation in the study, with differing amounts based on the questionnaire length and time required to complete the study. This QI study was designated as “Non-Human Subjects Research” by the Institutional Review Board of the Medical University of South Carolina.

### 2.2. Study design

**Figure 2:**
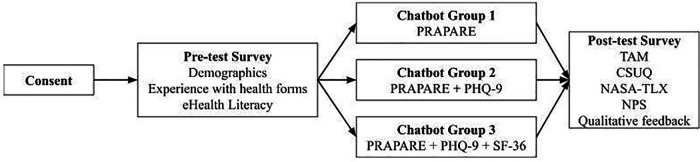
Study design.

This between-groups experimental design included a pre-test demographic questionnaire, health form completion using the chatbot, and a post-test semi-structured questionnaire (Figure 2). Our study was adapted from previous research investigating chatbot use among older adults (8). Prolific IDs were collected in pre- and post-test questionnaires to map participant responses throughout the study and assure data accuracy. Participants completed the forms in one sitting, or else the survey was timed-out by Prolific after 60 min.

*Step 1. Study information:* Participants were informed about the purpose and procedure of this QI study. Participants were ensured that their responses will be kept private and only used for research purposes. If interested, participants clicked a confirmation button to move forward with the study.
*Step 2. Pre-test questionnaire:* Participants completed questions on demographics, experience with health forms, and eHealth literacy (see Section 2.4.).
*Step 3. Chatbot-delivered health form:* Participants were redirected to a chatbot health form based on their random group assignment (see Section 2.3. for details about the health form groups).
*Step 4. Post-test questionnaire:* Once participants completed the chatbot-delivered health form, they were redirected to a post-test questionnaire where they were given several validated measures to assess perceptions of the chatbot (i.e., TAM, CSUQ, NASA-TLX, and NPS; see Section 2.4.) and answered two qualitative questions about their likes and dislikes of the chatbot (18–20).

### 2.3. Chatbot-delivered health forms

We developed three different groups of health forms to test the experience of chatbots at different form lengths. The health forms included the Protocol for Responding to and Assessing Patients' Assets, Risks, and Experiences (PRAPARE), the Patient Health Questionnaire (PHQ-9), and the Short Form-36 (SF-36) survey for quality of life (21–23). We chose these three health forms for the variety of question and response formats (e.g., text and numeric inputs, radio buttons, checkboxes) and broad applicability. See Table 1 for a summary of the health forms used in this study.

*Group 1 (Short; 21 Questions):* This group contained one form: PRAPARE. PRAPARE is a 21-item questionnaire to assess social drivers of health (21). For the standard PRAPARE question asking for a patient address, participants were instructed to input a fake address to protect their privacy. No personal identifying information was collected. All questions were required. Conversational steps included one-time components such as an introduction to the chatbot, fixed components such as question prompts, and occasional components such as motivating statements (e.g., “You are doing great” or “Keep it up, only 5 more questions to go!”). Participants in this group were asked to complete a total of 21 questions with 7 conversational steps.
*Group 2 (Moderate; 30 Questions):* Participants in this second group completed two health forms: PRAPARE and PHQ-9. PHQ-9 is a widely used 9-question form in behavioral and physical healthcare to assess mood (22). The chatbot workflow included an introduction, PRAPARE, PHQ-9, and motivating statements for a total of 30 questions and 8 conversational steps. All questions were required.
*Group 3 (Long; 66 Questions):* Participants in this third group completed three health forms: PRAPARE, PHQ-9, and SF-36. This 36-item form contains a variety of question types and is broadly applicable (23). The chatbot workflow included an introduction, PRAPARE, PHQ-9, SF-36, and motivating statements for a total of 66 questions and 14 conversational steps. All questions were required.

**Table 1:** Health forms used in the study.

Health form	Description	# of questions	Types of questions
PRAPARE	PRAPARE is a 21-item survey designed to measure individuals’ social determinants of health.	21	Text input, checkbox, yes/no, radio button, Likert scale
PHQ-9	The PHQ-9 is a 9-item validated, self-administrable tool to assess the severity of depression.	9	Radio button, Likert scale
SF-36	The SF-36 is a 36-item measure of quality of life and functional health and well-being from the patient's perspective.	36	Radio button, yes/no, Likert scale

PRAPARE, Protocol for Responding to and Assessing Patients Assets, Risks, and Experiences; PHQ-9, Patient Health Questionnaire-9; SF-36, Short Form-36.

### 2.4. Measures

#### 2.4.1. Demographics

We collected information on participant characteristics (i.e., age, sex, race, ethnicity, education) and previous experience completing health forms.

#### 2.4.2. eHealth literacy scale (eHEALS)

This is an 8-item eHealth literacy measure to assess combined knowledge, comfort, and perceived skills at finding, evaluating, and applying electronic health information to health problems (24). Responses are anchored on a five-point Likert Scale (i.e., 1 = Strongly Disagree to 5 = Strongly Agree).

#### 2.4.3. Technology acceptance model (TAM)

This 12-item measure based on the TAM is designed to assess perceived usefulness and ease-of-use, which are two fundamental determinants of user acceptance (18). Responses are anchored on a seven-point Likert Scale (i.e., 1 = Strongly Disagree to 7 = Strongly Agree).

#### 2.4.4. Computer system usability questionnaire (CSUQ)

The IBM CSUQ is a 19-item questionnaire designed to measure the perception of user experience (19). Responses are anchored on a seven-point Likert Scale (i.e., 1 = Strongly Disagree to 7 = Strongly Agree).

#### 2.4.5. NASA task load index (NASA-TLX)

This 6-item measure is designed to assess subjective mental workload in completing a task or using a system (20). The NASA-TLX comprises six sub-scales measuring mental, physical, and temporal demand, performance, effort, and frustration. Responses are anchored on a scale from 0 to 100, with a higher score indicating higher workload (i.e., 0 = Very Low to 100 = Very High). A NASA-TLX workload score of 13.08 is considered to be low, 46 as average, and 64.90 as high for cognitive tasks (25).

#### 2.4.6. Net promoter score (NPS)

The NPS is a 1-item measure of customer loyalty and likelihood to recommend a product and is considered a gold-standard rating of customer experience (26). In this study, participants were asked, “How likely are you to recommend Dokbot as a survey completion tool?”. Responses are anchored on a scale from 0 to 10 (i.e., 0 = Not at all likely to 10 = Extremely Likely). Individuals rating the product as 9 or 10 are considered promoters and 0–6 are considered detractors. The scores of 7 or 8 are considered passive scores and not included in NPS calculation. NPS is calculated by subtracting the percentage of detractors from the percentage of promoters and ranges between −100 to +100. Higher numbers of promoters entail a positive NPS score (≥0) representing higher enthusiasm and likelihood to recommend the product.

#### 2.4.7. Qualitative measures

Two open-ended questions asked participants about their likes (i.e., *What did you like about Dokbot*?) and dislikes (i.e., *What did you dislike about Dokbot*?) regarding the chatbot.

### 2.5. Data analysis

Descriptive measures were computed and included frequency, mean, median, and standard deviation. The eHEALS, TAM, CSUQ, NASA-TLX, and NPS scores were calculated according to standardized calculations. One-way analysis of variance tests were used to assess for statistically significant group differences in age and eHEALS scores with Bonferroni-corrected post-hoc comparisons. Analysis of covariance was used to compare group scores for TAM, CSUQ, NASA-TLX, and likelihood to recommend scores while covarying for age. MS Excel and IBM SPSS v28 were used for analyses.

Qualitative responses to open-ended questions were coded to identify emerging themes regarding the overall likes and dislikes of using the chatbot. Complete responses served as the units for coding. Content analysis was used to code participant responses into positive, negative, and neutral categories. Exploratory thematic analysis was completed by one researcher using MAXQDA qualitative data analysis software (27). A codebook was developed and refined by the research team over three iterations. Another researcher reviewed the codes, and any discrepancies were resolved through consensus among the team. Themes were quantified and organized by frequency and topic, which supplemented the quantitative analysis. Complex coding query, a type of qualitative analysis showing patterns of occurrence of previously coded data, can determine when certain themes or topics are commonly discussed together (sets). This type of analysis highlights complex, interrelated topics from thematic analysis and was used to identify what emergent themes were found in juxtaposition to tool opinions (28). The coding queries were conducted and verified by researchers. In our team, author HW conducted the complex coding queries and authors HS and JI reviewed the analysis.

## 3. Results

### 3.1. Participant characteristics

Of the 334 invited participants, 277 (82.9%) participants started the study and 260 (93.9%) participants completed the study in three groups (Group 1 = 92/113, 81.4%; Group 2 = 86/111, 74.5%; Group 3 = 82/110, 74.5%; see Table 2). Previous research assessing data quality of online crowdsourcing platforms suggests removal of fraudulent or duplicate responses, which were identified in the current study by examining and matching Prolific IDs between the pre-test and post-test questionnaires (16, 29). A total of 17 participants were excluded due to dropout (i.e., starting the pre-test but not completing the post-test; *n* = 12), duplicate Prolific ID (*n* = 3), and missing/incorrect Prolific ID (*n* = 2). No significant differences were found in the final sample sizes of the three groups [χ^2^(2, *N *= 260) = 0.75, *p *= 0.58].

**Table 2:** Study sample and excluded participants.

	Full sample	Group 1	Group 2	Group 3
Total participants invited	334	113	111	110
Participants who started the study	277	99	89	89
Participants who completed the study	260	92	86	82
Total excluded	17	7	3	7
Started but not completed	12	4	2	6
Duplicate Prolific ID	3	2	1	0
Missing/incorrect Prolific ID	2	1	0	1

The average age of participants was 67 years old, ranging from 60 to 93 years. Age significantly differed by group, *F*(2, 257) = 26.57, *p* < 0.001. Post-hoc tests revealed a significant difference among all groups (*p*s < 0.01). The participants were 51.2% female, 93.5% white, and 97.3% non-Hispanic. Participants had a bachelor's degree (36.5%), Master's degree (28.1%), or some college but no degree (14.6%). Participants' mean eHealth literacy score was 33.6 (SD = 4.7). eHealth literacy scores did not vary by group, *F*(2, 257) = 1.58, *p* = 0.21. Most (98.8%) participants reported that they had completed health forms before, commonly on paper (*n* = 241; 92.7%) and/or online (*n* = 200; 76.9%). See Table 3 for detailed characteristics.

**Table 3:** Participant demographics and experience with health forms questions.

The data are reported as *N* (%)	Full sample *N* = 260	Group 1 *N* = 92	Group 2 *N* = 86	Group 3 *N* = 82
**Age; *M* (SD)**	67.2 (5.1)	67.0 (4.5)	69.9 (3.9)	64.7 (5.5)
60–69	187 (71.9)	65 (70.7)	64 (74.4)	58 (70.7)
70–79	68 (26.2)	25 (27.2)	21 (24.4)	22 (26.8)
80+	5 (1.9)	2 (2.2)	1 (1.2)	2 (2.4)
**Sex**				
Female	133 (51.2)	49 (53.3)	41 (47.7)	43 (52.4)
Male	127 (48.8)	43 (46.7)	45 (52.3)	39 (47.6)
**Race**				
White	243 (93.5)	87 (94.6)	79 (91.9)	77 (93.9)
Black or African American	7 (2.7)	3 (3.3)	4 (4.7)	0 (0.0)
Native American or Alaskan Native	5 (1.9)	2 (2.2)	0 (0.0)	3 (3.7)
Asian	6 (2.3)	2 (2.2)	0 (0.0)	4 (4.9)
More than one race	3 (1.2)	2 (2.2)	1 (1.2)	0 (0.0)
Other	3 (1.2)	0 (0.0)	2 (2.3)	1 (0.2)
Native Hawaiian or other Pacific Islander	0 (0.0)	0 (0.0)	0 (0.0)	0 (0.0)
**Ethnicity**				
Hispanic, Latino, or Spanish origin	7 (2.7)	1 (1.1)	3 (3.5)	3 (3.7)
Not Hispanic/Latino	253 (97.3)	91 (98.9)	83 (96.5)	79 (96.3)
**Education**				
Middle school (grades 6–8)	1 (0.4)	0 (0.0)	0 (0.0)	1 (1.2)
High school graduate	21 (8.1)	9 (9.8)	5 (5.8)	7 (8.5)
Associate's degree	32 (12.3)	15 (16.3)	11 (12.8)	6 (7.3)
Some college (1–4 years, no degree)	38 (14.6)	13 (14.1)	13 (15.1)	12 (14.6)
Bachelor's degree	95 (36.5)	34 (37.0)	30 (34.9)	31 (37.8)
Master's degree or higher	73 (28.1)	21 (22.8)	27 (31.4)	25 (30.5)
**Experience with health forms**				
Yes	257 (98.8)	90 (97.8)	86 (100.0)	81 (98.8)
No	2 (0.8)	1 (1.1)	0 (0.0)	1 (1.2)
I don’t know	1 (0.4)	1 (1.1)	0 (0.0)	0 (0.0)
**Health forms approaches**				
On paper, at home or doctor's office	241 (92.7)	80 (87.0)	81 (94.2)	80 (97.6)
Online, at home or at doctor's office	200 (76.9)	69 (75.0)	67 (77.9)	64 (78.0)
Verbally, with someone's help	10 (3.8)	4 (4.3)	2 (2.3)	4 (4.9)
Verbally, at doctor's office	51 (19.6)	14 (15.2)	19 (22.1)	18 (22.0)
**eHealth Literacy; *M* (SD)** ^a^	33.6 (4.7)	33.9 (5.2)	32.9 (4.7)	34.1 (4.2)

^a^ Cronbach's alpha = .93.

### 3.2. Perceived ease-of-use and usefulness

Older adults reported a mean perceived ease-of-use score of 5.8 (SD = 1.1) and a mean perceived usefulness score of 4.7 (SD = 1.7; Table 4). There was a statistically significant difference in perceived usefulness among groups, *F*(2, 256) = 3.08, *p* = 0.048, *η*_p_^2^ = 0.02. Pairwise comparisons revealed that the mean perceived usefulness rating for Group 1 was significantly higher than the mean rating for Group 3 (*p *= 0.01, 95% CI = [0.13, 1.14]). There was no statistically significant difference among groups in perceived ease-of-use, *F*(2, 256) = 2.01, *p* = 0.14, *η*_p_^2^ = 0.02.

**Table 4:** Measures scores, *M* (SD**).**

Measure	Score Range	Full Sample	Group 1	Group 2	Group 3
**TAM**					
Perceived Usefulness^a^	1–7	4.7 (1.7)	5.0 (1.5)	4.7 (1.7)	4.3 (1.8)
Perceived Ease-of-use^b^	1–7	5.8 (1.1)	5.8 (1.0)	6.0 (1.0)	5.6 (1.2)
**CSUQ** ^c^					
Overall Score	1–7	5.4 (1.3)	5.5 (1.1)	5.6 (1.2)	5.1 (1.5)
System Usefulness	1–7	5.8 (1.3)	5.9 (1.2)	6.0 (1.2)	5.5 (1.6)
Information Quality	1–7	5.1 (1.3)	5.2 (1.2)	5.2 (1.2)	4.8 (1.4)
Interface Quality	1–7	5.3 (1.5)	5.5 (1.3)	5.4 (1.4)	5.0 (1.8)
**NASA-TLX**					
Overall workload (Raw, unweighted)	0–100	12.3 (12.1)	12.0 (10.9)	11.5 (11.9)	13.4 (13.4)
Mental demand	0–100	18.4 (19.5)	16.9 (16.6)	17.7 (20.1)	20.8 (21.9)
Physical demand	0–100	6.8 (11.0)	6.6 (8.5)	7.8 (14.0)	5.9 (9.8)
Temporal demand	0–100	13.9 (19.9)	14.8 (20.5)	13.7 (19.0)	13.1 (20.4)
Performance	0–100	8.5 (20.6)	10.2 (23.5)	5.7 (13.3)	9.4 (23.3)
Effort	0–100	13.4 (18.8)	11.8 (15.5)	14.1 (20.6)	14.5 (20.3)
Frustration	0–100	12.8 (24.3)	11.8 (21.2)	10.0 (19.6)	16.9 (30.9)
**Net Promoter Score**	−100 to 100	0	5	6	−12

^a^ Cronbach's alpha = .98.

^b^ Cronbach's alpha = .86.

^c^ Cronbach's alpha = .97.

### 3.3. Chatbot system usability

Table 4 shows that older adults reported a mean usability score of 5.4 (SD = 1.3). There were no statistically significant differences in CSUQ total scores among groups, *F*(2, 256) = 2.28, *p* = 0.10, *η*_p_^2^ = 0.02. Further, there were no statistically significant differences among groups in scores for system usefulness (*F*[2, 256] = 2.11, *p* = 0.12, *η*_p_^2^ = 0.02), information quality (*F*[2, 256] = 1.83, *p* = 0.16, *η*_p_^2^ = 0.01), and interface quality (*F*[2, 256] = 2.59, *p* = 0.08, *η*_p_^2^ = 0.02).

### 3.4. Cognitive load

Participants’ responses for the NASA-TLX showed low overall mental workload (*M* = 12.3; SD = 12.1; Table 4). There were no statistically significant differences in the total NASA-TLX score among groups, *F*(2, 256) = 0.11, *p* = 0.90, *η*_p_^2^ = 0.001. There were also no statistically significant differences among groups for the NASA-TLX subscales of mental demand (*F*[2, 256] = 0.65, *p* = 0.52, *η*_p_^2^ = 0.01), physical demand (*F*[2, 256] = 0.39, *p* = 0.68, *η*_p_^2^ = 0.003), temporal demand (*F*[2, 256] = 0.32, *p* = 0.73, *η*_p_^2^ = 0.002), performance (*F*[2, 256] = 0.52, *p* = 0.60, *η*_p_^2^ = 0.004), effort (*F*[2, 256] = 0.59, *p* = 0.55, *η*_p_^2^ = 0.005), or frustration (*F*[2, 256] = 1.01, *p* = 0.37, *η*_p_^2^ = 0.01).

### 3.5. Likelihood to recommend

Groups 1 and 2 reported positive NPS of 5 and 6 respectively (Table 4). The NPS declined among Group 3 participants to −12. Although the scores declined for Group 3, no significant differences were observed in reported likelihood to recommend the chatbot among the three groups, *F*(2, 256) = 2.17, *p *= 0.12, *η*_p_^2^ = 0.02.

### 3.6. Qualitative analysis of chatbot likes and dislikes

Table 5 represents common themes, definitions, and example responses related to the theme. Most of the comments were positive (69.0%) with some negative (23.6%) and neutral (7.3%).

**Table 5:** Themes and codebook: older adults’ likes and dislikes about chatbots.

Micro category	# of codes	Definition	Illustrative examples
**Macro Category: User Descriptors**			
Ease-of-use	157	Discussion of ease of filling out the health form, including being simple, clear, straightforward, concise, efficient, user friendly, and intuitive.	*“Seemed easy and straightforward to use.*”
Completion time	48	Statements related to how long it took to complete the health form.	*“It was quick to take my answer and move on.*”
Humanness	28	Reference to chatbot feeling personable or impersonal.	*“It was just like chatting with a human*”
Positive Experiences	22	Comments about the overall positive experience (e.g., interactive, engaging, fun, nice, pleasant, friendly).	*“I liked the “friendliness*” *of the dokbot.*”
**Macro Category: Interface Quality**			
Design	50	Design or formatting of the chatbot interface.	*“The screen was a little too little.*”
Navigation	43	Transitions between questions and overall cadence of the interaction.	*“Advances automatically after answering.*”
Technology Experience	25	Technology-related experiences or problems.	*“It didn't always advance automatically. Sometimes it showed the answers, but the question was obscured, by the previous question, so I had to advance it using the scroll bar.*”
Actionable Suggestions	23	Improvement suggestions for the chatbot interface or survey.	*“If I needed to change an answer, I could not go back through the form.*”
Comparison with other data collection approaches	19	Comparison of chatbot experience to other survey intake formats (e.g., online form, pen and paper, doctor, etc).	*“Much easier than filing* [sic] *out forms by hand and it is rather fun.*”
Progress	19	Progress updates and motivating statements such as “Great job!”.	*“It wasted a little time with some greats or just a few more questions. I would rather just see a progress bar or % completion*”
Privacy	15	Discussion of feelings of safety (or lack of) when answering questions and providing personal health information.	*“I'm always a little hesitant to provided* [sic] *personal or medically related information online to someone I don't know. Prefer in-person or on the phone in those type of matters. Otherwise I had no issues with the dokbot.*”
**Macro Category: Miscellaneous**			
Survey Specific	85	Comment specific to the survey completed in the study, not related to chatbot delivery	*“Questions about my living situation and health*”

The category *ease-of-use* had the greatest number of codes (*n*= 157) with 100% positive codes. Participants commonly referred to ease-of-use in understanding and answering questions, perceiving the chatbot experience as *easy* (*n* = 109/157 codes), *clear*/*straightforward* (*n* = 38/157 codes), and *simple* (*n* = 25/157 codes). One participant said, “*easy to use, quick and convenient.*” Several participants noted “*simple and straightforward*” or “*quick and to the point.*” With regard to completion time, most participants commented on the quickness of the form, but participants also noted “*it takes much longer than doing it myself*” and “*too slow, needless interaction.*” Participants referenced a human-like aspect of interacting with the chatbot, making neutral to positive comments such as “*very easy to communicate with, felt like talking with a real person, expressed warmth and empathy*” (*n* = 19/28 codes). On the other hand, some participants felt the chatbot was “*impersonal and reactionless*” or that it “*lacked a personal touvh* [sic]” (*n *= 9/28 codes).

Participants commonly noted *positive experiences* (*n* = 22 codes) of using the chatbot, saying it was “*fun,*” “*nice,*” “*pleasant,*” and “*friendly.*” One participant commented, “*I thought the questions were brief and understandable. I thought filling in the forms felt almost fun doing them with the dokbot. I felt I could be honest without judgement* [sic] *with dokbot.*” Several participants commented on the friendliness of the chatbot, writing responses such as “*I liked that it seemed friendly, like a person.*”

*Technology experience* had mostly negative (*n* = 12/25 codes; 48.0%) to neutral (*n* = 11/25 codes; 44.0%) comments, citing problems with chatbot functioning, for example, “*it didn’t always advance automatically. Sometimes it showed the answers, but the question was obscured by the previous question, so I had to advance it using the scroll bar.*” They commented on the experience of using the technology, reporting some technical issues and privacy concerns, and some participants provided actionable suggestions for enhancements to the survey and chatbot interface such as sound, more personalized dialogue, and a way to ask questions or clarify answers.

*Design* had 28% positive responses (*n *= 14/50 codes) with participants commenting that “*it was easy to understand and has a gentle graphic interfac*e” and “*I liked the bright colors and the friendly feel of it. It was also very clear and I liked the font.*” There were several neutral comments (*n *= 6/50 codes; 12%) about *design*, such as “*I wonder how they would respond to my concerns.*” Participants expressed that they would like to change “*size of the bot and the font*” or noted that “*the screen was a little too little*” (*n *= 30/50; 60%).

Participants overall found the *navigation* to be pleasant and seamless, commenting that it was “*smooth and uncomplicated*” and that “*it moved along at a good pace.*” The chatbot dialogue included intermittent progress updates and positive encouragement such as “Awesome! You are doing great!” Participants shared mixed views about these encouraging messages, with some expressing like and others dislike of the messages. Of the 19 times that it was mentioned, 5 were positive, 12 were negative, and 2 were neutral. Participants commented “*I was less thrilled when it wrote things like ‘you are doing really well!’. It seemed a bit condescending*” or “*I liked how easy it was to answer the questions and how the questions were asked, plus the encouragement such as ‘you’re doing great.*”

The *comparison with other data collection approaches* code received 8/19 (42.1%) neutral to positive comments and 57.9% (*n* = 11/19 codes) negative responses. Participants compared the chatbot to other survey experiences such as talking to a nurse/doctor and paper or online forms. Some participants enjoyed “*not having to talk to a person*” while others “*would have preferred a person.*” Further, some noted it was “*much easier than filing* [sic] *out forms by hand and it is rather fun*” but “*if you had a problem or were confused, it may be easier to talk to a person.*”

Participants commented on *privacy* concerns with providing personal information to the chatbot. This category had the majority of negative comments (*n* = 10/15 codes; 66.7%) with some participants finding the chatbot “*a bit intrusive.*” One participant commented, “*I’m always a little hesitant to provided* [sic] *personal or medically related information online to someone I don’t know. Prefer in-person or on the phone in those type of matters. Otherwise I had no issues with the dokbot.*” Other (*n* = 5/15 codes; 33.3%) participants stated that the chatbot exhibited mannerisms similar to their providers, commenting, “*It seemed pleasant. It stated questions clearly, and sensitively. It didn’t rush me.*” Another participant mentioned that the chatbot “*asked probing questions just like a doctor would.*”

#### 3.6.1. Complex coding query by group

Complex coding query was performed to identify group differences in qualitative responses (Table 6). In Group 1, 76.5% (*n* = 117/153) of codes were positive, 7.8% (*n* = 12/153) were neutral, and 15.7% (*n* = 24/153) were negative. In Group 2, 68.7% (*n* = 103/150) of codes were positive, 3.3% (*n* = 5/150) were neutral, and 28.0% (*n* = 42/150) were negative. In Group 3, 61.6% (*n* = 90/146) of codes were positive, 11.0% (*n* = 16/146) were neutral, and 27.4% (*n* = 40/146) were negative. Comments specific to the survey questions (such as “*Questions about my living situation and health*”) were excluded (85 codes) from the positive, negative, and neutral coding categories displayed in Table 5.

**Table 6:** Complex coding query of positive, negative, and neutral responses and themes (*N* = 449).

Code	Positive				Neutral				Negative			
	Group 1 (*N* = 117)	Group 2 (*N* = 103)	Group 3 (*N* = 90)	Total (*N* = 310)	Group 1 (*N* = 12)	Group 2 (*N* = 5)	Group 3 (*N* = 16)	Total (*N* = 33)	Group 1 (*N* = 24)	Group 2 (*N* = 42)	Group 3 (*N* = 40)	Total (*N* = 106)
Ease-of-use	56	58	43	157	0	0	0	0	0	0	0	0
Completion time	21	13	12	46	0	0	0	0	0	1	1	2
Navigation	17	9	11	37	0	0	0	0	0	2	4	6
Positive experiences	5	8	9	22	0	0	0	0	0	0	0	0
Humanness	6	6	4	16	2	0	1	3	3	4	2	9
Design	5	5	4	14	2	0	4	6	6	9	15	30
Comparison with other data collection approaches	4	1	1	6	1	0	1	2	2	6	3	11
Progress updates	1	2	2	5	0	1	1	2	3	7	2	12
Privacy	0	1	2	3	1	1	0	2	6	3	1	10
Technology experience	1	0	1	2	3	3	5	11	3	5	4	12
Actionable suggestions	1	0	1	2	3	0	4	7	1	5	8	14

Group 3 had the greatest number of negative codes in *design*, *actionable suggestions*, and *navigation* categories. Participants commented on the chatbot color scheme, size of the screen, limited chat function, and font. Further, with regard to *navigation*, participants commented that the movement “*was a little ‘jerky’ and unnerving, and could be smoother.*” Group 1 had the most positive codes for *completion time* (“*quick*,” “*fast*,” “*brief*”), *navigation* (“*flowed nicely*”), and *compare* (“*it was just like chatting with a human*”). Group 2 had the most negative codes for *humanness* (“*impersonal and reactionless*”), *compare* (“*the fake interaction made giving the information take longer than just filling out a form would have taken; the dokbot added absolutely no value*”), *progress update*s (“*dialogue a little too ‘cutesy’*”), and *technology experience* (“*seemed awkward—like I had to keep scrolling it up to see the questions*”).

## 4. Discussion

### 4.1. Main findings

As health technologies are increasingly incorporated into practice, it is important to make designs and user experiences friendly to older adults. In this QI study of experiences with a HDC chatbot (Dokbot), older adults found the chatbot-delivered health forms to be easy, useful, usable, and required low cognitive load. We identified opportunities to reduce concerns regarding design, privacy, and technical issues that would improve overall user experiences.

Participants reported a mean perceived ease-of-use score of 5.8/7 with correspondingly positive qualitative feedback. The ease-of-use theme had the greatest number of codes (157/449) with older adults frequently commenting that it was easy to understand and answer the questions using the chatbot. We observed no group differences in perceived ease-of-use, meaning older adults found the chatbot easy to use regardless of length of health forms. It was noted that scores were higher for perceived ease-of-use than perceived usefulness. The conversational design and encouraging statements provided by the chatbot may have felt engaging for shorter forms but became more tedious in longer forms. Although participants often compared the chatbot to talking with a human, older adults mentioned preferring to speak to a real person to ask for clarifications with the forms. However, this also holds true for other remote approaches such as online questionnaires and paper-based forms. In the future, the ability to ask questions to discuss with their providers at a later time could potentially alleviate these concerns. Future research should further examine the timing and structure of motivating statements, comparing generic praise (e.g., “Great job! Keep it up”) against more goal-oriented messaging (e.g., “This information helps your doctor understand your treatment progress. Keep going!”).

Participants reported a mean usability score of 5.4/7 with 54.8% (*n* = 51/93) providing positive comments for navigation and design. The chatbot was designed to show one question at a time, advancing automatically when the participant answered a question. This design maintained steady progress and may have provided a good user experience, but this automated design and small interface may have been more difficult for some participants to read or review responses across the form. Previous research found that participants praised chatbots for being “*quick and easy*” despite taking longer to complete (28, 30). The humanlike, conversational, and pleasant design of the chatbot may take longer but may also make it easier or more enjoyable to complete the health forms. However, this finding could be due to the absence of a comparative medium against the chatbot.

In a 2015 study, Grier et al. reported that a NASA-TLX workload score of 13.08 is considered to be low, 46 to be average, and 64.90 as high for cognitive tasks (25). Average NASA-TLX scores were low (12.3/100) in the current study, which suggests older adults required low cognitive load to complete the chatbot. Considering the low mental demand, chatbot HDC may provide a better experience for older adults if they experience challenges with memory or cognitive decline (31). Some features of the chatbot may contribute to this finding, as the chatbot does not rely on working memory to answer questions. It is notable that mental demand scores are somewhat higher than other subscales, which could be because health form questions can be specific and require close reading and concentration. Future research could improve older adults' experience by improving accessibility and readability of chatbot-delivered health forms.

Our findings echo previous studies that have evaluated chatbot-delivered health forms. Ponathil et al. (2020) evaluated age differences in perceptions of chatbot-delivered family health history forms using TAM, CSUQ, and NASA-TLX, reporting older adults preferred a chatbot over the standard interface for family health history collection despite taking longer to complete (8). The study showed older adults reported high perceived usefulness, ease-of-use, and satisfaction for the chatbot. Another study found most participants preferred a chatbot (Dokbot) to an online form (REDCap) even though the chatbot took longer to complete (28). Participants in this study perceived the chatbot as easy to use and feeling as though they were talking to a human with over 69% positive comments, showing a positive attitude towards the chatbot. Participants echoed comments about the easy navigation and structural flow, noting that elements such as answering one question at a time eased their worries about skipping a question.

It is notable that some older adults commented about security concerns or feeling that the chatbot was “*intrusive*” (*n* = 15 codes). There were several comments relating to the trust, safety, and privacy of using the chatbot for data collection of protected health information. Previous researchers have commented that older adults have concerns about data privacy, which we observed as well with participants expressing concerns about the chatbot invading their privacy, not being connected to a medical authority, and reservations providing medical information to an unfamiliar source (11, 32). This worry could be due to misconceptions about how the data is being used upon collection. Future research may look at the situational impact of where data is collected. Older adults may be less concerned about security if they engaged with the chatbot for HDC in a familiar, trusted healthcare setting (e.g., white labeling with their own provider's information). These concerns could also be alleviated with more information about chatbot security, HIPAA-compliance, and relation to a trusted medical professional. Future research should examine strategies to describe data security policies and precautions in order to maximize patient confidence in automated HDC.

A secondary goal of this study was to assess participant's experience of completing chatbot-delivered health forms with three different lengths. Results show a significant difference in perceived usefulness between Group 1 (*M* = 5.0) and Group 3 (*M* = 4.3), meaning older adults' belief that the chatbot enhanced their performance declined as the length increased. We also noticed a decline in NPS to −12 (Group 3) from 5 (Group 1) and 6 (Group 2), reflecting that there were more detractors than promoters in Group 3. The scores were lower in comparison with a previous study with a majority younger (95.1% participants <60 years) population (chatbot NPS = 24). Negative comments also increased with more concerns about design and formatting as the length of the chatbot increased from 6 (Group 1) to 9 (Group 2) to 15 (Group 3). These findings point toward favorable user experiences with shorter chatbots but poorer experiences over longer interaction with the chatbot. Factors such as restricted content space, lack of progress tracking or time estimate, and the constrained presentation of questions one-by-one could hinder a user's experience over a longer form. Future research should further explore the impact of adaptive chatbots that alter variables such as length of forms, types of questions, progress tracking, and speech patterns to personalize user experiences.

Participants also provided actionable suggestions to improve the chatbot usability and accessibility for older adults. Most participants provided various suggestions to enhance interfaces and text formatting to increase readability (e.g., font size, color, text editing, etc.). Participants also commented on the inability to edit a previous response. Although undoing previous responses was available in the chatbot, the feature may not have been salient in use. Participants also desired features such as voice capabilities (such as voice-to-text data entry or text-to-voice for reading chatbot questions), viewing a summary of their responses before submission, easy inputs, and ability to ask for clarifications.

### 4.2. Limitations

Participants were recruited using an online crowd-sourcing platform, Prolific, which may not be representative of the general population. We aimed to recruit participants who were 60 years or older but made no further specifications for race or education. Participants might be more technologically savvy compared to the general population considering their presence on the platform, which may have contributed to high reported eHealth literacy scores (33–35). A previous study testing reliability and validity of eHEALS reported mean scores of 30.94 ± 6.00 among 866 older adults, which is somewhat lower than our sample mean of 33.6 ± 4.7 (36). Future studies should include larger, diverse groups (e.g., accommodating for race, technology experience, internet access, income, education) to better understand older adults' experience and accessibility needs. We note that participants were asked in the survey ‘what is your gender?’ but correct wording should have specified ‘sex” rather than “gender”.

Due to a technology issue with the chatbot and inability to track time between different technologies, we were unable to collect time data for the study and chatbot-delivered health form. Future studies should look into assessing time taken to complete chatbot health forms and user experience.

Individuals may have completed the questionnaires inaccurately or disingenuously considering the remote, unmoderated nature of the study. Individuals could have completed the chatbot-delivered health form and questionnaires in a hurry or at their own convenience and provided careless responses. Although, researchers have reported on the high quality of data collected using Prolific suggesting that data quality may not be a concern (16). Future research should consider conducting moderated studies to directly observe participants as they complete experimental chatbot arrangements.

Further, biases can occur from paid survey pools for remote, unmoderated studies, specifically at low levels of compensation (37). Framing bias is possible due to the crowdsourcing recruitment approach; however, such a framing effect is more likely seen regarding questions related to money and risk–topics not considered in this study.

## 5. Conclusion

The study presents findings that chatbots could be a valuable modern HDC approach for older adults. Older adults reported chatbot-delivered health forms to be easy, useful, and usable, and additionally, to require low cognitive load. They reported overall positive experiences and ease-of-use of the chatbot and concerns about technology issues, privacy, and the lack of ability to ask clarifying questions. Many of the participants' responses lead to actionable suggestions such as focusing on design, accessibility, and privacy. As the length of the survey increased, older adults reported a decrease in perceived usefulness, likelihood to recommend, and an increase in negative comments. Improvements in chatbot design and features may make them a useful, interactive data collection tool for health forms of varying length. Findings have broad implications for HDC and chatbot development, warranting continued investigation to establish best practices and design recommendations for the older adult population.

## Data Availability

The raw data supporting the conclusions of this article will be made available by the authors, without undue reservation.

## Footnotes

^1^ https://dokbot.io/

^2^ https://www.prolific.co/
